# Giant Pseudoaneurysm as an Uncommon Late Complication Following a Fourteen-Year Femoropopliteal Bypass in a Visually Impaired Patient

**DOI:** 10.3390/diagnostics16050686

**Published:** 2026-02-26

**Authors:** Emil-Marian Arbănași, Cristian Trâmbițaș, Constantin Claudiu Ciucanu, Réka Bartus, Eliza-Mihaela Arbănași, Paul Mateica, Timea Madaras, Marius Mihai Harpa, Adrian Vasile Mureșan, Eliza Russu

**Affiliations:** 1 Department of Vascular Surgery, George Emil Palade University of Medicine, Pharmacy, Science and Technology of Targu Mures, 540139 Targu Mures, Romania; emil.arbanasi@umfst.ro (E.-M.A.); claudiu.ciucanu@umfst.ro (C.C.C.); reka.kaller@umfst.ro (R.B.); adrian.muresan@umfst.ro (A.V.M.); eliza.russu@umfst.ro (E.R.); 2 Clinic of Vascular Surgery, Mures County Emergency Hospital, 540136 Targu Mures, Romania; paulmateica@yahoo.com; 3 Regenerative Medicine Laboratory, Centre for Advanced Medical and Pharmaceutical Research (CCAMF), George Emil Palade University of Medicine, Pharmacy, Science and Technology of Targu Mures, 540139 Targu Mures, Romania; arbanasi.eliza@gmail.com; 4 Doctoral School of Medicine and Pharmacy, George Emil Palade University of Medicine, Pharmacy, Science and Technology of Targu Mures, 540139 Targu Mures, Romania; timea.madaras@umfst.ro; 5 Department of Plastic and Reconstructive Surgery, George Emil Palade University of Medicine, Pharmacy, Science and Technology of Targu Mures, 540139 Targu Mures, Romania; 6 Department of Surgery No. IV, George Emil Palade University of Medicine, Pharmacy, Science and Technology of Targu Mures, 540139 Targu Mures, Romania; mihai.harpa@umfst.ro

**Keywords:** peripheral arterial disease, vascular surgery, synthetic graft, complications, multidisciplinary approach, pseudoaneurysm, bypass

## Abstract

**Background:** Non-anastomotic pseudoaneurysm formation due to very late prosthetic graft failure after femoropopliteal bypass is exceptionally rare. **Case Presentation:** We describe a 73-year-old blind man who presented with rapid enlargement of a mid-thigh mass on the left side, associated with skin necrosis. His history included advanced atherosclerosis with bilateral superficial femoral artery occlusion and prior femoropopliteal bypasses: a right-sided great saphenous vein graft (2006) and a left-sided Dacron^®^ graft (2008). Computed tomography angiography revealed a giant pseudoaneurysm originating from the mid-portion of the left bypass graft (13.8 × 16.5 cm) with active contrast extravasation and distal popliteal artery occlusion, as well as a large, well-defined lateral thigh lipoma. Open surgery revealed structural graft disruption within the prosthetic body and a large chronic pseudoaneurysm sac containing organized thrombus. En bloc pseudoaneurysm excision and graft exclusion without reconstruction were performed, followed by soft-tissue reconstruction. The postoperative course was uneventful, with complete wound healing by four weeks and no ischemic symptoms during 18 months of follow-up. This exceptionally late prosthetic graft complication underscores the need for long-term surveillance in patients with lower-limb bypass grafts. **Conclusions:** This case highlights that prosthetic graft failure may occur very late and present insidiously. Recognition of this rare complication is essential for timely diagnosis and individualized surgical management.

## 1. Introduction

Peripheral arterial disease (PAD) is defined as a reduction in peripheral artery perfusion, which occurs due to atherosclerotic deposits within the arterial system [1,2]. Based on the extent of atherosclerotic involvement, patients diagnosed with PAD are categorized according to the Rutherford classification in six distinct stages: asymptomatic individuals (stage 0), those experiencing mild to moderate claudication (stage I/II), patients with severe claudication (stage III), individuals suffering from rest pain (stage IV), patients presenting with digital trophic lesions (stage V), and those with severe ischemic ulcers or gangrene (stage VI) [1].

Endovascular treatment has emerged as the primary option for patients suffering from PAD with short occlusions or severe arterial stenoses in the femoropopliteal region [3,4,5,6]. In contrast, patients with extensive occlusions are advised to consider surgical revascularization [7,8,9]. Consequently, for those patients identified as suitable candidates for femoropopliteal bypass, autologous vein grafts are favored over prosthetic grafts due to their demonstrably superior long-term patency rates [10,11]. Nevertheless, prosthetic grafts may serve as a viable alternative for revascularization in cases where the saphenous vein is inaccessible [12,13]. In contrast, Zlatanovic et al. [14] noted that bypass surgery exhibits lower rates of re-intervention in comparison to percutaneous transluminal angioplasty, with or without stenting (PTA/S), over the long term for GLASS grade III and IV femoropopliteal lesions (*p* = 0.002). Graft rupture constitutes the rarest long-term complication associated with synthetic grafts, often accompanied by the formation of pseudoaneurysms and bleeding episodes [15,16].

This manuscript aims to present the step-by-step surgical treatment of a giant pseudoaneurysm that emerged atypically, occurring fourteen years after an above-the-knee femoropopliteal bypass in a visually impaired patient with a history of several prior revascularization procedures.

## 2. Case Presentation

**Figure 1:**
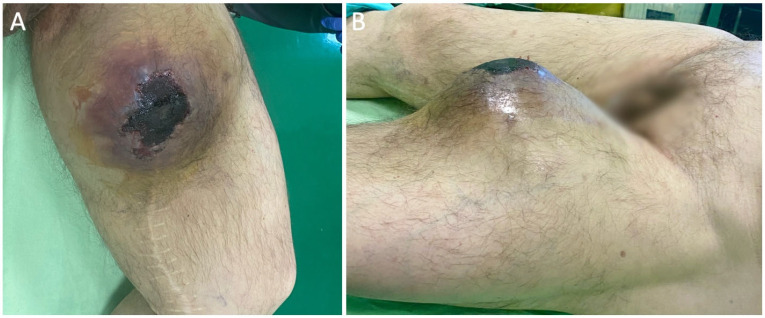
Clinical macroscopic aspect of the medial thigh demonstrating a large, tense soft-tissue swelling with overlying skin changes. (**A**) Anteromedial view showing a well-demarcated formation with central hemorrhagic necrosis/eschar. (**B**) Lateral view illustrating marked focal protrusion and skin tension over the medial thigh.

We present the case of a 73-year-old male blind patient who presented to the emergency department due to an alarming growth of a mass in the middle third of the left thigh associated with skin necrosis (Figure 1). The patient’s medical history encompasses generalized atherosclerosis, characterized by bilateral superficial femoral artery occlusion and the presence of bilateral femoropopliteal bypass procedures—utilizing the great saphenous vein in the right limb in 2006 and a Dacron^®^ graft in the left limb, performed in 2008. Additionally, the medical history includes arterial hypertension, ischemic heart disease, chronic heart failure, and a history of tobacco use. Preoperative laboratory evaluation demonstrated no pathological findings. There were no systemic indicators of infection, and both leukocyte count and C-reactive protein levels were within normal limits.

**Figure 2:**
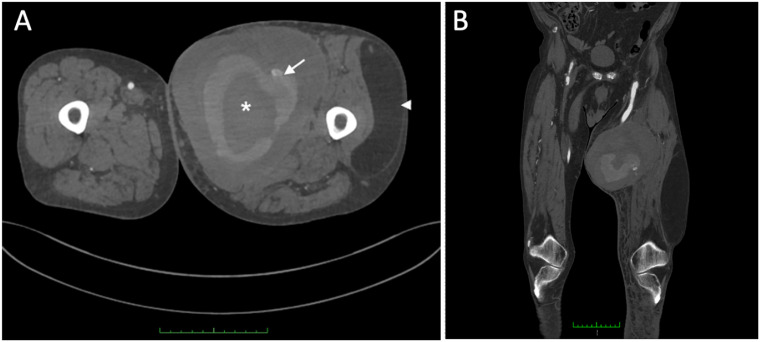
Cross-sectional (**A**) and coronal (**B**) images of the thighs demonstrating a large pseudoaneurysm (asterisk) developing around a synthetic above-knee femoropopliteal bypass graft, with active contrast extravasation from the graft (arrow) and a giant lipoma (arrowhead).

Ultrasound demonstrated a patent left femoropopliteal bypass coursing through the large thigh mass, prompting further assessment with computed tomography angiography (CTA). CTA revealed a giant pseudoaneurysm involving the mid-portion of the bypass prosthesis, measuring 13.8 × 16.5 cm (AP × LL), causing marked expansion of the adjacent soft tissues. Active contrast extravasation from the prosthesis was evident (Figure 2A), and the popliteal artery was occluded at the P3 segment. In addition, a giant lipoma was identified in the lateral thigh compartment, measuring 10.8 × 5.0 × 22.1 cm (AP × LL × CC), with a well-circumscribed, homogeneous fat density (Figure 2A). The coronal reconstruction (Figure 2B) depicts the full craniocaudal extent of both the pseudoaneurysm and lipoma and illustrates their relationship to the femoropopliteal graft and adjacent musculature.

**Figure 3:**
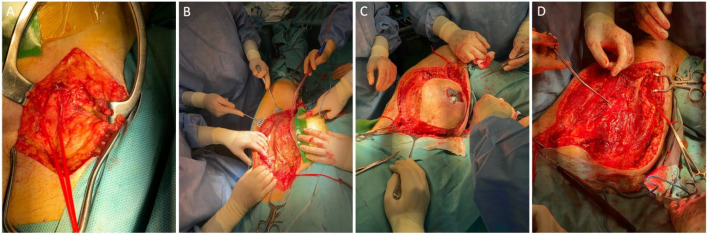
Intraoperative photographs illustrating the main sequential steps of the procedure. (**A**) Initial exposure following skin incision with subcutaneous dissection to access the proximal portion of the synthetic graft, which is secured with silicone loops. (**B**) Progressive dissection around the pseudoaneurysm to the level of the distal anastomosis to allow bleeding control and vascular clamping. (**C**) Careful, meticulous dissection with mobilization of the pseudoaneurysm. (**D**) Final operative view after en bloc resection of the giant pseudoaneurysm and closure of the proximal and distal anastomoses.

An open surgical intervention was chosen for the en bloc exclusion of the formation without a vascular reconstruction because of the popliteal artery occlusion and higher risk of infection (Figure 3). Thus, the procedure begins with exposure of the proximal portion of the previously implanted synthetic graft, which is encircled and controlled with a silicone loop (Figure 3A). Dissection is then carefully extended around the pseudoaneurysm, progressing toward the distal anastomosis to obtain precise vascular control and enable safe clamping (Figure 3B). With proximal and distal control secured, meticulous circumferential mobilization of the pseudoaneurysm is performed, safeguarding adjacent structures and minimizing blood loss (Figure 3C). After full mobilization, the pseudoaneurysm is excised en bloc, revealing the underlying anastomotic sites, which are then directly closed with Prolene 5-0 (Figure 3D).

**Figure 4:**
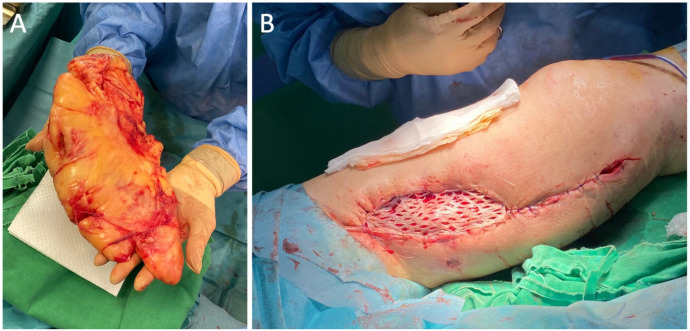
Intraoperative photographs demonstrate: (**A**) a large specimen after resection, featuring a sizable lobulated adipose tissue mass with surrounding soft tissue, and (**B**) the recipient site of the internal thigh after resection of the pseudoaneurysm, covered with a meshed split-thickness skin graft and secured with sutures.

The plastic surgery team proceeded with excision of the giant lipoma in the lateral thigh compartment using the same surgical approach. This facilitated mobilization of the adjacent skin and allowed closure of the defect with a meshed split-thickness skin graft, which was secured with sutures (Figure 4). A drainage tube was also inserted at the site of the giant lipoma removal and further removed on the third postoperative day.

**Figure 5:**
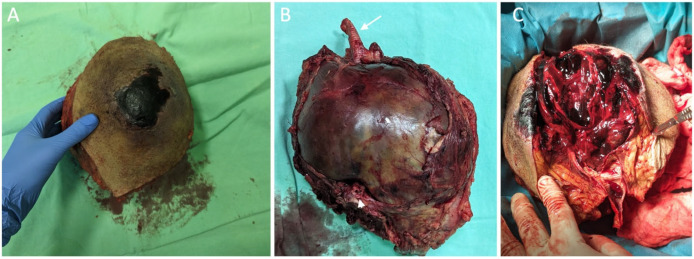
Macroscopic appearance of the resected giant pseudoaneurysm. (**A**) External surface of the specimen showing an elliptical skin paddle with central cutaneous necrosis overlying the pseudoaneurysm. (**B**) Deep surface demonstrating the tense, fibrotic pseudoaneurysm sac; the proximal portion of the synthetic vascular graft is indicated by the arrow and the distal portion by the arrowhead. (**C**) Specimen opened longitudinally, revealing the pseudoaneurysmal cavity with graft disruption, organized intraluminal thrombus, and laminated clot adherent to the inner wall.

Following en bloc excision of the large pseudoaneurysm, gross inspection revealed a thick, fibrotic pseudoaneurysm sac enveloping segments of the synthetic vascular graft (Figure 5A,B). Longitudinal opening of the specimen demonstrated a pseudoaneurysmal cavity filled with organized thrombus and laminated clot, features consistent with a chronic lesion associated with graft disruption (Figure 5C). Intraoperative specimens obtained from the pseudoaneurysm sac were sent for microbiological analysis. Culture results identified *Escherichia coli*, and the antibiotic therapy treatment was initiated. Despite the absence of systemic signs of infection and normal inflammatory markers, this finding raised suspicion of a possible low-grade graft infection contributing to structural graft failure.

**Figure 6:**
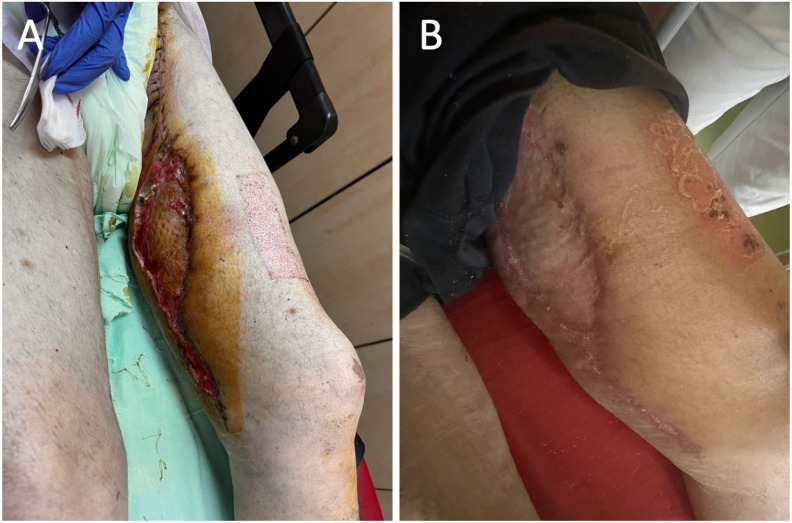
Postoperative clinical course of the patients: (**A**) two-week follow-up showing an extensive full-thickness soft-tissue defect of the lateral thigh with a healthy granulation tissue bed, and (**B**) four-week follow-up of the same region demonstrating complete re-epithelialization and scar formation.

Postoperatively, during the two-week follow-up, evidence of successful skin graft integration was noted (Figure 6A). Additionally, at the four-week follow-up visit, complete healing of both the donor and skin graft sites was observed (Figure 6B). Moreover, the patient reported no lower-limb ischemic symptoms during the 18-month follow-up period, with complete healing of both donor and skin graft sites.

## 3. Discussion

This case highlights an exceptionally rare late complication following above-the-knee femoropopliteal bypass with a synthetic graft, characterized by progressive graft failure and the formation of a giant pseudoaneurysm 14 years after implantation. It underscores that prosthetic graft rupture may occur very late after surgery and can present insidiously as a progressively enlarging thigh mass with overlying skin compromise, rather than with acute hemorrhage or ischemia. Advanced imaging with CTA was essential for diagnosis, anatomic delineation, and operative planning, while definitive multidisciplinary management required individualized open surgical treatment with en bloc resection and soft-tissue reconstruction. This case, therefore, emphasizes the need for sustained long-term surveillance of prosthetic bypasses.

PAD remains a major cause of morbidity worldwide, with over a 70% increase in global prevalence from 1990 to 2019 [17,18]. Unfortunately, approximately 55% of patients diagnosed with PAD may be attributed to usual risk factors such as active smoking, diabetes, and hypertension [17]. The femoropopliteal segment is the most frequently affected site of atherosclerotic occlusive disease and often requires repeated endovascular or open surgical interventions [19]. When feasible, autologous vein grafts are preferred because of their superior long-term patency; however, prosthetic conduits such as Dacron^®^ or PTFE are commonly employed when suitable venous conduits are unavailable [17,18,19]. Although complications of prosthetic bypass grafts—including thrombosis and infection—are well recognized, late graft rupture with pseudoaneurysm formation is uncommon, particularly beyond ten years after implantation [20,21,22,23,24,25,26].

One study reported the histological characteristics of Dacron^®^ and polytetrafluoroethylene (PTFE) vascular grafts explanted from patients after 4 to 20 years in vivo [20]. The authors observed typical foreign-body reactions and fibrotic healing within the interstices of Dacron^®^ grafts and on the external surfaces of both Dacron^®^ and PTFE grafts. No signs of resorption or biodegradation were detected in any specimen. In addition, a pseudointimal layer was identified on the luminal surface of Dacron^®^ grafts but was absent in PTFE grafts [20]. More recently, several meta-analyses comparing these two prosthetic materials have been published. Two of these studies reported no significant difference in outcomes for above-the-knee femoropopliteal bypass procedures [21,22], whereas another concluded that Dacron^®^ grafts demonstrated superior performance [23]. Consistent with this, van Det et al. [24] reported superior patency rates for Dacron^®^ grafts in a cohort of 228 above-the-knee femoropopliteal bypasses followed for up to 10 years.

The pathogenesis of late pseudoaneurysm formation after prosthetic bypass is multifactorial [25,26,27]. Mechanical fatigue of the graft material, repetitive motion in areas of stress, low-grade chronic infection, and anastomotic degeneration have all been implicated as contributory mechanisms [25,26,27]. In the present case, disruption within the mid-graft segment rather than at the anastomoses suggests intrinsic structural degradation of the prosthetic material, which is extremely rarely reported in the literature. The presence of a large, organized intraluminal thrombus supports a chronic, progressive pattern of enlargement. The patient’s visual impairment likely contributed to delayed presentation, underscoring how vulnerable or disabled individuals may develop substantial, neglected lesions before seeking medical care.

Nevertheless, it is important to recognize that this complication can still occur, most commonly as a result of local factors. In patients undergoing femoropopliteal bypass surgery, the development of a pseudoaneurysm—particularly in the setting of graft infection—constitutes a serious, albeit relatively uncommon, late complication that can adversely affect long-term outcomes. Graft infection is a major contributing factor, occurring in approximately 2.6% of patients [28]. When infection undermines the integrity of the graft–artery interface, bacterial enzymatic activity and inflammatory tissue degradation can weaken the suture line and adjacent structures, thereby increasing the risk of anastomotic pseudoaneurysm formation [29].

The European Society of Vascular Surgery (ESVS) guidelines recommend routine postoperative imaging surveillance after infrainguinal bypass—most commonly using duplex ultrasound (DUS)—to enable early detection of clinically silent graft stenoses and allow timely intervention, thereby reducing the risk of graft thrombosis and limb loss [2]. However, despite these recommendations for mid- and long-term surveillance, contemporary evidence remains inconsistent, raising questions regarding the real-world clinical benefit of systematic DUS follow-up [30,31,32]. Stocco et al. [30] reported improved limb salvage and survival among patients enrolled in a DUS surveillance program, although less than 60% completed the scheduled follow-up, highlighting the challenge of long-term adherence. In contrast, Dar et al. [31] observed a reduction in overall mortality associated with DUS surveillance, but no significant impact on amputation rates, suggesting that improved survival may not necessarily translate into enhanced limb-related outcomes. Furthermore, Koo et al. [32] demonstrated low compliance with surveillance protocols and identified non-adherence as a predictor of vein graft occlusion, whereas no similar association was observed for prosthetic grafts. Collectively, these findings suggest that while DUS surveillance has the potential to improve clinical outcomes, its effectiveness is highly dependent on patient adherence and graft type, underscoring the need for more targeted surveillance strategies and optimized follow-up protocols in routine clinical practice.

Pseudoaneurysms arising at non-anastomotic sites are distinctly uncommon, with only sporadic cases reported in the literature [33,34,35]. When they do occur, they are most frequently associated with post-traumatic injury at the site of a vascular graft or develop as a late complication secondary to chronic mechanical fatigue, material degradation, or repetitive biomechanical stress—particularly in anatomical regions exposed to sustained motion or high hemodynamic forces. Aldermir et al. [33] reported successful surgical management of a rare para-anastomotic femoral artery pseudoaneurysm in a 57-year-old patient, identified five years after femoropopliteal bypass surgery. Similarly, Selçuk et al. [34] described a thrombosed, non-anastomotic aneurysm of a biosynthetic graft in a patient who had undergone femoropopliteal bypass using an Omniflow II graft three years earlier. Bensaid et al. [35] documented a particularly unusual presentation involving dual non-anastomotic pseudoaneurysms following axillofemoral bypass with an ePTFE graft. Across all reported cases, management consisted exclusively of open surgical intervention, with strategies focused on pseudoaneurysm exclusion and bypass graft reconstruction when patency was preserved at the time of diagnosis.

This case illustrates that prosthetic femoropopliteal bypass grafts remain susceptible to structural failure and pseudoaneurysm formation even beyond a decade after implantation, challenging the assumption that late mechanical complications are negligible. It emphasizes the necessity for sustained, protocol-driven postoperative surveillance, as very late graft disruption may evolve insidiously and manifest only after significant local tissue compromise. From a clinical management perspective, early recognition through imaging and individualized surgical decision-making are pivotal to preventing life-threatening hemorrhagic or limb-threatening sequelae.

## 4. Conclusions

This case presents an exceptionally rare, gigantic pseudoaneurysm resulting from failure of the mid-portion of a Dacron^®^ femoropopliteal bypass 14 years after its implantation, complicated by overlying skin necrosis and an adjacent large lipoma in a patient with visual impairment. It demonstrates that very late graft rupture, although uncommon, must be considered in the differential diagnosis of thigh masses in patients with prior bypass surgery. The rarity and delayed presentation of this complication underscore the need for heightened clinical vigilance and prolonged surveillance in patients with prosthetic lower-limb bypasses.

## Data Availability

Data are available upon request.

## Abbreviations

The following abbreviations are used in this manuscript:

PAD	peripheral arterial disease
PTA/S	percutaneous transluminal angioplasty, with or without stenting
CTA	computed tomography angiography
PTFE	polytetrafluoroethylene
